# Pharmacogenomic Biomarkers in US FDA-Approved Drug Labels (2000–2020)

**DOI:** 10.3390/jpm11030179

**Published:** 2021-03-04

**Authors:** Jeeyun A. Kim, Rachel Ceccarelli, Christine Y. Lu

**Affiliations:** 1 Department of Epidemiology, Harvard T.H. Chan School of Public Health, Boston, MA 02115, USA; jeeyunkim@mail.harvard.edu; 2 Department of Population Medicine, Harvard Pilgrim Health Care Institute and Harvard Medical School, Landmark Center, 401 Park Drive, Suite 401 East, Boston, MA 02215, USA; rachel.ceccarelli@umassmemorial.org

**Keywords:** pharmacogenomics, precision medicine, US Food and Drug Administration, clinical actionability

## Abstract

Pharmacogenomics (PGx) is a key subset of precision medicine that relates genomic variation to individual response to pharmacotherapy. We assessed longitudinal trends in US FDA approval of new drugs labeled with PGx information. Drug labels containing PGx information were obtained from Drugs@FDA and guidelines from PharmGKB were used to compare the actionability of PGx information in drug labels across therapeutic areas. The annual proportion of new drug approvals with PGx labeling has increased by nearly threefold from 10.3% (*n* = 3) in 2000 to 28.2% (*n* = 11) in 2020. Inclusion of PGx information in drug labels has increased for all clinical areas over the last two decades but most prominently for cancer therapies, which comprise the largest proportion (75.5%) of biomarker–drug pairs for which PGx testing is required. Clinically actionable information was more frequently observed in biomarker–drug pairs associated with cancer drugs compared to those for other therapeutic areas (*n* = 92 (59.7%) vs. *n* = 62 (40.3%), *p* < 0.0051). These results suggest that further evidence is needed to support the clinical adoption of pharmacogenomics in non-cancer therapeutic areas.

## 1. Introduction

Approximately 80% of the variability in drug efficacy and adverse effects can be explained by genomic variation, which creates major challenges for the appropriate selection and dosing of medications [1]. Genomic composition is an important factor for individual response to therapy by affecting the expression of drug targets, drug metabolizing enzymes, and other proteins involved in pathophysiological mechanisms pertaining to the drug’s pharmacodynamic and pharmacokinetic processes [2,3]. As an applicable component of precision medicine, pharmacogenomics (PGx) incorporates genomic profiling to identify biomarkers based on relevant genotype–phenotype interactions that can predict drug response and risk of adverse drug reactions for individual patients. Novel next-generation sequencing techniques have enabled rapid growth of PGx knowledge, with over 200 genome-wide association studies (GWAS) of pharmacotherapy responses reported to date [4].

In particular, key somatic variants, such as the overexpression of *ERBB2* in breast cancer, can serve as markers for the selection of patient groups for which drugs like ado-trastuzumab emtansine and talazoparib tosylate are indicated. Pharmacogenomic testing for germline variants, such as those in the *DPYD* gene, can also predict the risk of toxicity and differential response to cancer therapies such as 5-fluorouracil, enabling prescribers to better tailor therapies with greater efficacy and safety for patients [5,6]. In other areas, PGx biomarkers have been used to specify dosing alterations, as in the example of the *CYP2C9* and *VKORC1* variants in warfarin use for treatment and prevention of thromboembolic events, as well as to prevent the occurrence of severe hypersensitivity effects, as with an *HLA-B* variant in relation to abacavir use for treatment of HIV infection [3,7].

Recent advances in the development of targeted therapies against specific variants have increased the efficiency of clinical trials by enabling smaller trial sizes, higher success rates, and expedited time to market [8,9,10,11]. Over the last two decades, the US Food and Drug Administration (FDA), the agency responsible for reviewing and approving a drug for marketing if it provides benefits that outweigh its known potential risks, has actively encouraged the incorporation of genomic data into drug development, including the issuance of several guidelines for industry regarding submission of PGx information as part of the drug review process [12]. This study examined trends in FDA approvals of new drugs labeled with PGx information from 2000 to 2020. We anticipated that the proportion of new drug approvals with PGx labeling would increase over time. We also compared the level of clinical actionability of biomarker information in drug labels across various therapeutic areas.

## 2. Materials and Methods

### 2.1. Data Extraction and Evaluation

All data came from publicly available sources on the FDA and PharmGKB websites. Initial drug and biologic approval reports from 1 January 2000 to 31 July 2020 were gathered from the Drugs@FDA database [13]. We extracted the following information in a standardized format from Drugs@FDA: drug brand name, active ingredient, approval date, submission classification, and therapeutic area. Among drug approvals with duplicate active ingredients, we included only the drug with the earliest initial approval date to our study. A drug can have several approval dates: one for each application submitted for FDA review. Among all retrieved entries, new drug applications (NDAs) with “Type 1 New Molecular Entity” and “Type 1/4 New Molecular Entity and New Combination” with the corresponding approval dates were examined in order to capture drug labels at the time of first approval. Because this classification does not exist for biologics, the earliest approval date and drug label were used for biologics. For drugs indicated for multiple diseases, we considered only the therapeutic areas that were relevant to the biomarker information.

Drug labels were reviewed for PGx information based on the FDA Table of Pharmacogenomic Biomarkers in Drug Labeling (called FDA Table hereafter) and the PharmGKB website; we included a biomarker for a drug if it was listed by either source [14]. The FDA Table lists approved products with PGx information in the drug labeling and specifies sections of the labeling that contained biomarker information [14]. The PharmGKB is a publicly available knowledgebase for PGx that provides annotations of medication prescribing guidelines based on published evidence for gene–drug associations [15]. We compared and verified the names of the listed biomarkers from both sources with the information provided in the first-approved drug labels extracted from Drugs@FDA. Since a drug can have multiple biomarkers and a single biomarker can be labeled for more than one drug product, we counted the number of unique biomarker–drug pairs mentioned in the first-approved drug label only; we did not include biomarkers included in labeling updates.

### 2.2. Drug Label Annotations of PGx Levels

PharmGKB provides four types of annotations (PGx Levels) for PGx information associated with specific gene–drug combinations:

1. “Required genetic testing”, where labels state or imply that gene, protein, or chromosomal testing, including genetic testing, functional protein assays, cytogenetic studies, should be conducted prior to using the drug. Testing may only be required for a subset of patients. A label that states that the variant is an indication for the drug or that a test “should be performed” is also interpreted as requiring testing;
2. “Recommended genetic testing”, where labels state or imply that gene, protein, or chromosomal testing, including genetic testing, functional protein assays, cytogenetic studies, is recommended prior to using the drug. The recommendation may only be for a subset of patients. A label that states that testing “should be considered” or “consider genotyping or phenotyping” is also considered to recommend testing;
3. “Actionable PGx”—marked for labels that describe the impact of gene/protein/chromosomal variants or phenotypes on changes in efficacy, dosage, metabolism, or toxicity, including mention of contraindication of the drug in a subset of patients defined by particular variants/genotypes/phenotypes. However, labels with this annotation do not require or recommend gene, protein, or chromosomal testing;
4. “Informative PGx”—assigned to labels that state particular gene/protein/chromosomal variants or metabolizer phenotypes do not affect a drug’s efficacy, dosage, metabolism, or toxicity, or that variants or phenotypes affect a drug’s efficacy, dosage, metabolism, or toxicity, but this effect is not clinically significant. This level is also assigned to all other labels that have been listed in the FDA Table but do not currently meet the criteria for all other PharmGKB PGx annotations listed above [15].

We considered PGx information to be clinically actionable if they were categorized as “required genetic testing,” “recommended genetic testing,” or “actionable PGx.” Biomarker–drug pairs were considered to lack actionability if they were assigned an “informative PGx” level by PharmGKB; examples of “informative” biomarker–drug pairs include those with labels that only describe the role of a variant in the drug’s metabolism or state that dose adjustment or other actions were not necessary for a particular variant.

### 2.3. Statistical Analysis

Descriptive statistics were used to characterize trends in approval rates of new pharmacogenomic drugs and compared the clinical actionability of PGx information in drug labels between cancer therapies and drugs used in non-cancer therapeutic areas. We performed Fisher’s exact tests to determine if PGx testing requirements and overall clinically actionable information were more frequently associated with cancer biomarker–drug pairs compared to biomarkers for all other clinical areas.

## 3. Results

Of 694 total new drug approvals identified from 1 January 2000 to 31 July 2020, new molecular entities accounted for 75.9% (*n* = 527) and biologics represented 24.1% (*n* = 167). Biosimilars comprised 16.8% (*n* = 28) of newly approved biologics. On average, there were about 33 new approvals per year and they ranged from a minimum of 18 drug approvals in 2007 to a maximum of 66 approvals in 2018. Cancer therapies comprised 23.1% (*n* = 160) of total drug approvals.

About a quarter of total new drug approvals (25.6%; *n* = 178) contained PGx biomarker information in initial approved labels. An estimated 74.7% (*n* = 133) of approvals with PGx labeling were for new molecular entities while the remaining 25.3% (*n* = 45) were for biologics. Biosimilars accounted for 15.6% (*n* = 7) of biologics approved with PGx labeling and 3.9% of overall initial drug approvals with PGx labels.

**Figure 1:**
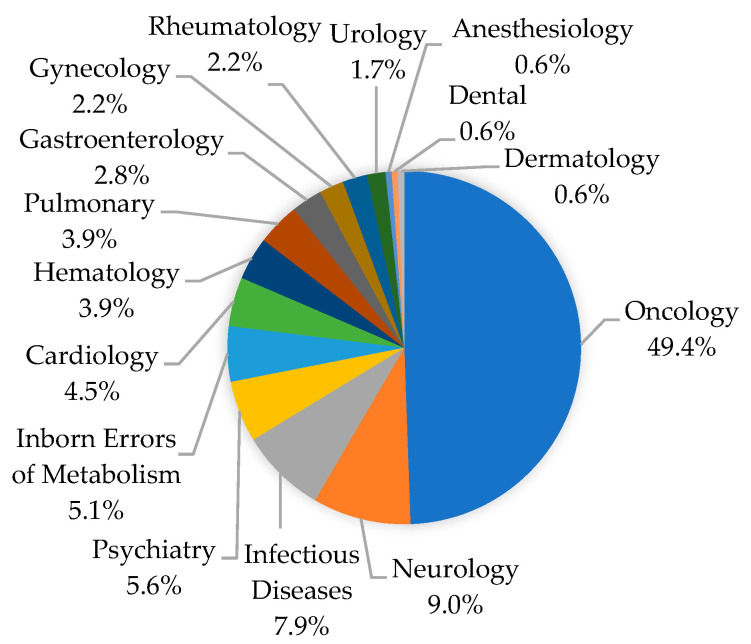
Therapeutic areas of new FDA drug approvals with pharmacogenomics (PGx) labeling from 2000 to 2020.

Figure 1 shows the distribution of therapeutic areas for 178 drug approvals with PGx labeling. Oncology was the most common therapeutic area, comprising 49.4% (*n* = 88) of all new drugs approved with PGx labeling. Other therapeutic areas included neurology (*n* = 16; 9.0%), infectious diseases (*n* = 14; 7.9%), psychiatry (*n* = 10; 5.6%), inborn errors of metabolism (*n* = 9; 5.1%), cardiology (*n* = 8; 4.5%), hematology (*n* = 7; 3.9%), and pulmonology (*n* = 7; 3.9%). The remaining clinical areas that each comprised less than 3% of PGx labels were gastroenterology, gynecology, rheumatology, urology, anesthesiology, dentistry, and dermatology.

### 3.1. Yearly Trends in Drug Approvals with PGx Information

**Figure 2:**
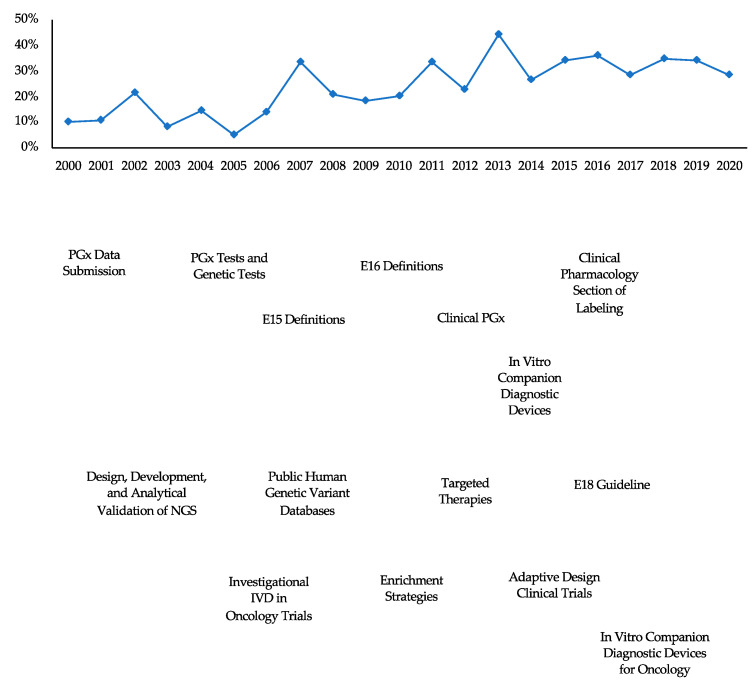
Trends in annual proportion of new drug approvals with PGx labeling and finalized regulatory guidance related to pharmacogenomics from 2000 to 2020, with data through 31 July 2020. E15, E16, and E18 Guidance were developed within the International Conference on Harmonisation of Technical Requirements for Registration of Pharmaceuticals for Human Use (ICH) and endorsed by the ICH Steering Committee at Step 4 of the ICH process [16].

**Figure A1:**
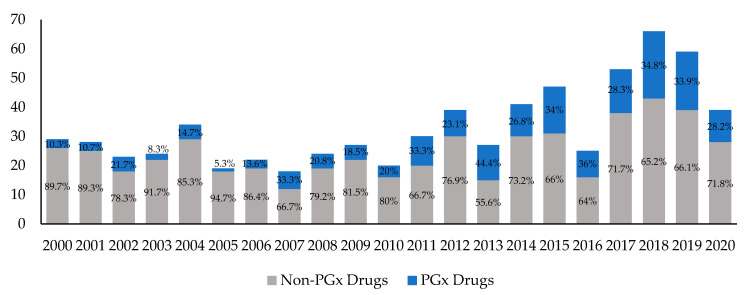
Trends in the number of new drug approvals with PGx labeling from 2000–2020. Data shown through July of 2020.

Overall, the average proportion of new drug approvals with PGx labeling was 23.8% per year from 2000 to 2020. The annual proportion of new drug approvals with PGx labeling increased by approximately threefold from 10.3% (*n* = 3) in 2000 to 33.9% (*n* = 20) in 2019 and 28.2% (*n* = 11) through July in 2020; with the lowest at 5.3% (*n* = 1) in 2005 and highest at 44.4% (*n* = 12) in 2013 (Table 1, Figure A1). This growth is emphasized in the latter half of the study period, during which there has also been a proliferation of regulatory guidance documents related to PGx (Figure 2).

Among cancer drugs, the average proportion of drug approvals with PGx labeling was 52.0% per year from 2000 to 2020. The annual proportion of new cancer drug approvals with PGx labeling increased from 33.3% (*n* = 1) in 2000 to 55.6% (*n* = 10) in 2019 and 47.1% (*n* = 8) through July in 2020 with the lowest at 0% in 2008 and highest at 100% (*n* = 4) in 2016 (Table 1).

Among non-cancer drugs, the average proportion of drug approvals with PGx labeling was 16.2% per year from 2000 to 2020. The annual proportion of new non-cancer drug approvals with PGx labeling increased from 7.7% (*n* = 2) in 2000 to 24.4% (*n* = 10) in 2019 and 13.6% (*n* = 3) through July in 2020 with the lowest at 0% in 2005 and highest at 38.9% (*n* = 7) in 2013 (Table 1).

### 3.2. Yearly Trends of Biomarker–Drug Pairs

Forty-three (24.2%) drugs of all drug approvals with PGx labeling contained multiple biomarkers at initial approval, with a maximum of 7 biomarkers in one drug label, resulting in a total of 258 unique biomarker–drug pairs identified from 2000 to 2020. Of these, 52.3% (*n* = 135) were for cancer indications. On average, there were 12.3 biomarker–drug pairs approved per year over the study period. The number of biomarker–drug pairs approved annually increased from 3 in 2000 to 35 in 2019 and 17 through July of 2020 with a minimum of 1 in 2005 and maximum of 43 in 2018.

**Figure 3:**
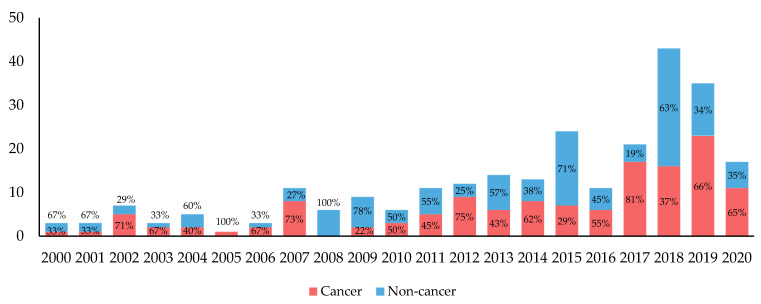
Trends in the number of new biomarker–drug pairs approved per year with annual proportions by cancer vs. non-cancer from 2000 to 2020. Data shown through July of 2020.

The average annual proportion of biomarker–drug pairs indicated for cancer was 53%. Between 2000 and 2020, the annual proportion of biomarker–drug pairs with cancer indications increased from 33.3% (*n* = 1) in 2000 to 66% (*n* = 23) in 2019 and 65% (*n* = 11) in 2020 with the lowest at 0% in 2008 and highest at 100% (*n* = 1) in 2005 (Figure 3).

For the remaining 123 non-cancer biomarker–drug pairs, the average annual proportion of biomarker–drug pairs was 47%. The annual proportion of biomarker–drug pairs with indications for all other clinical areas decreased from 67% (*n* = 2) in 2000 to 34% (*n* = 12) in 2019 and 35% (*n* = 6) in 2020 with the lowest at 0% in 2005 and highest at 100% (*n* = 6) in 2008 (Figure 3).

### 3.3. Clinical Actionability of PGx Information

**Figure 4:**
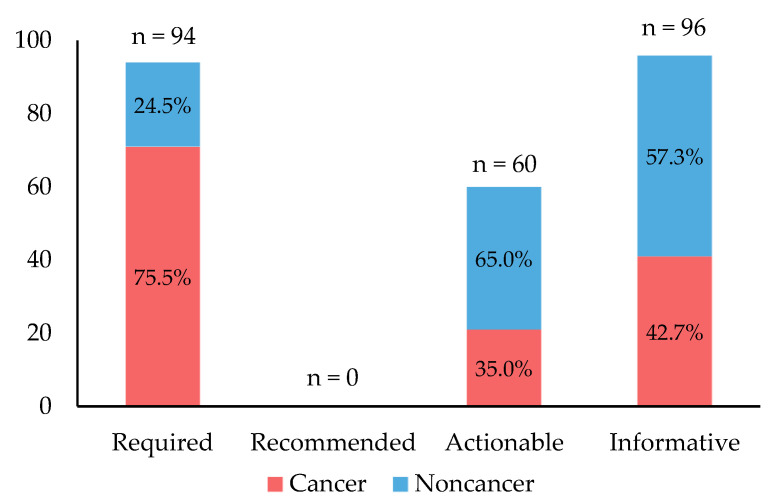
PharmGKB PGx Levels of biomarker–drug pairs for cancer and non-cancer therapies.

Figure 4 depicts the distribution of biomarker–drug pairs across PGx levels of drug label information based on PharmGKB categories. Of 250 biomarker–drug pairs annotated with PGx levels, 61.6% (*n* = 154) are clinically actionable; of these, 59.7% (*n* = 92) were associated with cancer drugs while the remaining 40.3% (*n* = 62) were associated with drugs for non-cancer areas (*p* < 0.0051). Biomarker–drug pairs considered to be clinically actionable included 37.6% (*n* = 94) of total biomarker–drug pairs that require genetic testing (cancer accounted for 75.5% while non-cancer accounted for 24.5%; *p* < 0.0001), 0% recommend genetic testing, and 24.0% (*n* = 60) correspond to “actionable” information (cancer accounted for 35.0% while non-cancer accounted for 65.0%). The remaining 38.4% (*n* = 96) of biomarker–drug pairs were “informative” (cancer accounted for 42.7% and non-cancer 57.3%) but lacked clinical actionability.

## 4. Discussion

With recent progress in genomic sciences and precision medicine along with regulatory guidance for pharmacogenomics, it is not surprising that we found PGx biomarkers have become increasingly prevalent in new drug labels for all therapeutic areas over the last two decades. Greater than half of all biomarker–drug pairs identified in our study were associated with clinically actionable measures of PGx information. Consistent with previous studies, this progress has continued to be most prominent in cancer therapies, which comprise the majority of new PGx drug approvals and account for the greatest proportion of biomarker–drug pairs with testing requirements [17,18,19].

In 2005, the FDA issued its first guidance for industry with information on how to submit PGx data during new drug application and review processes, including specific uses of PGx information in drug labeling [20]. Recommendations for co-development of new drugs and corresponding companion diagnostic devices (i.e., PGx tests), in the absence of available tests, were included in the initial document, with further guidance on in vitro diagnostics development published in 2014, 2019, and 2020 [21,22,23]. The majority of PGx-related guidance for industry, such as guidelines for including genetic information into appropriate sections of the labeling and the creation of the “Pharmacogenomics” subsection within the Clinical Pharmacology section, were issued in the latter half of the study period. There has been a corresponding substantial increase in the number of new drug approvals with PGx information in the labeling, with a total of 136 new drugs between 2011 and 2020 compared to 42 new drugs between 2000 and 2010 [24,25].

Cancer drugs have maintained a strong presence in PGx, as well as among targeted therapies in general. Greater knowledge of clinically significant gene–drug interactions (particularly in relation to somatic variants) has in part enabled the prediction of treatment efficacy in targeted patient subgroups and prompted industry investment in biomarker-based strategies for novel cancer drug development. A review of FDA-approved cancer therapies that required PGx testing demonstrated that two-thirds of drug approvals were based on an enrichment trial design [26]. Such trial designs have been associated with greater clinical trial success rates and lower costs associated with drug development, particularly for well-validated biomarkers such as *HER2* for the treatment of metastatic breast cancer [8,9,10,11]. Recent studies have further attributed improvements in cancer survival to the approval of several new PGx-based targeted treatments for metastatic cancer [27,28].

Targeted approaches to immunotherapy have recently changed the landscape of therapeutic strategies in cancer. For instance, immune-checkpoint inhibitors act against checkpoint protein (PD-1) or its partner protein (PD-L1), enabling the activation of an anti-tumor immune response. Identifying predictive biomarkers for checkpoint blockade response is critical for optimizing treatment efficacy and preventing drug-related toxic effects. These inhibitors have been associated with improved survival and fewer adverse events compared with chemotherapy for various tumor types, including metastatic melanoma, advanced non-small cell lung cancer (NSCLC), and head and neck squamous cell carcinoma [29,30]. Molecular diagnostics have been approved for the use of anti-PD-1/anti-PD-L1 therapies, particularly for pembrolizumab, nivolumab, and atezolizumab [31]. Several other biomarkers are also promising for predicting immunotherapy response. High tumor mutation burden identifies tumors with a greater number of variants that may be more easily recognized by the immune system, which has been correlated with benefit from anti-PD-1/anti-PD-L1 therapies for cancers such as melanoma and NSCLC [32]. Mismatch repair deficiency/microsatellite instability is a predictor of anti-PD-1/anti-PD-L1 treatment efficacy in solid tumors such as colorectal cancer [33,34]. Human leukocyte antigen (HLA) is another promising biomarker as it plays a major role in discerning foreign pathogens or tumor cells as part of the anti-tumor immune response [35]. Research suggests that a patient’s HLA type might be indicative of response to immunotherapy and can be utilized in personalized cancer vaccine development and immunotherapy biomarker discovery [36].

The use of PGx is also important outside of cancer but their application may be limited by the following: the availability of other established biomarkers, such as blood pressure, hemoglobin A1C, and low-density lipoprotein used to assess patient prognostic risk in cardiovascular disease clinical trials, and the complexity of drug metabolism, particularly for conditions such as chronic kidney disease that can alter drug response phenotypes (e.g., phenoconversion) [7,37,38].

PGx-guided therapies in non-cancer areas (e.g., cardiovascular disease, mental illness) have primarily focused on the cytochrome P450 (CYP) family of pharmacogenes, which are involved in the metabolism of nearly 20% of commonly used drugs [39]. For example, variation in the *CYP2D6* gene influences drug metabolism activity such that individuals who carry deficient *CYP2D6* alleles have sub-optimal enzymatic activity and are at higher risk of developing adverse drug reactions. Among psychotropic medications, there are several drug substrates for *CYP2D6*, including atomoxetine (attention deficit hyperactivity disorder medication) and clozapine (antipsychotic for treatment of schizophrenia), for which drug labels recommend dose adjustments for patients who are *CYP2D6* poor metabolizers [40]. Studies provide accumulating evidence that PGx testing for CYP enzyme genes can inform drug dosing and selection and improve patient outcomes [41,42,43,44].

The vast majority of actionable drug labels with testing requirements provided genotype-based indication or contraindication. Although PGx labeling could help inform physicians determine an appropriate treatment plan for a patient, its impact on clinical practice may be hindered by the following considerations: the amount of evidence available to support the pharmacologic relevance of genomic associations is highly variable at the time of labeling [45,46]; and PGx information in some drug labels are informational only [46,47,48]. An example is the drug label for lenalidomide, which mentions a specific PGx variant as part of the indication but does not explicitly require testing prior to drug use. Other labels provide information on the impact of the variant on drug response without recommending a clinical action (e.g., the label for fesoterodine stated that a subset of individuals are poor metabolizers for *CYP2D6* and “Cmax and AUC of the active metabolite are increased 1.7- and 2-fold, respectively, in *CYP2D6* poor metabolizers, as compared to extensive metabolizers” [49]. The clinical significance of these increased concentrations was not stated, and it implies that prescribers need to order PGx testing for *CYP2D6* for some patients and modify the dosage according to individual genotype status).

In addition to PGx levels, PharmGKB provides clinical annotations of variant–drug associations that are assigned with “level of evidence” scores using several criteria to measure the confidence in the association based on literature findings such as replication of association, *p* value, and odds ratio [50]. The PharmGKB Clinical Annotation Levels of Evidence have been used to support other relevant guidelines, such as CPIC Levels for Genes/Drugs, which summarize literature-based evidence, strength of prescribing recommendation, and the corresponding clinical context for the use of PGx information in drug labels [51]. The degree of consistency between the levels of evidence and strength of prescribing recommendations is presently unclear and may be an area for further research. Notwithstanding, the application of actionable PGx information may depend on a range of other factors which may be context-dependent and subjective in nature. Hendricks-Sturrup et al. outlined several scenarios highlighting considerations such as therapeutic alternatives, timing of PGx testing with respect to diagnosis, and patient medical history and family history that influence decision-making for either incorporating or excluding certain PGx tests as part of patient management [52].

Our analysis suggests that we are likely to see continued growth in the prevalence of new drugs approved with PGx information, albeit with greater actionability for cancer treatment compared to all other clinical areas. We agree with the earlier commentary of Tutton that while actionable PGx information can help inform prescribing decisions, the increased approval of drugs containing PGx biomarkers serves only a partial role in facilitating large-scale adoption of PGx [18]. Additional challenges to the clinical adoption of PGx testing have been indicated in the literature, including the dearth of evidence supporting the clinical utility and cost-effectiveness of PGx testing [53,54,55]. Most salient of these is the underrepresentation of non-European ancestry in GWAS used to examine PGx traits and in clinical drug trials, which may result in ambiguity in the interpretation of PGx biomarkers for non-European patients and contribute to potential disparities in the utilization of PGx testing in cases where they are required [56,57]. A study conducted by Lynch et al. reported underutilization of guideline recommended PGx testing (e.g., *EGFR* testing in lung cancer) and substantial differences in the likelihood of getting tested based on the patient’s race as well as other demographic factors, including socioeconomic status and zip code [58]. As the number of new drugs with actionable PGx information continues to expand, further research is needed to address the ethical and social implications of the current Eurocentric bias in pharmacogenomic research to ensure equitable benefit of PGx for all members of society.

There were several limitations related to the data analyzed. We studied only the approvals of new drug applications with a submission classification of Type 1 or Type 1,4 and initial submissions for biologics. Approvals of generic drug products and applications with other NDA classification codes for already marketed active ingredients were not assessed, such that new biomarkers added to labels in the post-market setting or included as part of approvals through other NDA classification codes were excluded from our study. Our rationale for focusing solely on these submission types was to assess the incorporation of PGx information in initial drug approvals. We also did not account for biomarkers associated with approved drugs that have been discontinued or labels that were changed due to reports of unexpected adverse effects or failure to verify clinical benefit.

We observed differences in drugs considered to have PGx labeling between PharmGKB and the FDA Table. At the time of our study, 11 drugs with PGx labeling corresponding to 16 unique biomarker–drug pairs were profiled in PharmGKB but were not listed in the FDA Table. Reasons for discrepancies in PGx biomarkers listed by these sources are unclear but may be attributed to different criteria for annotation of PGx biomarkers. Conversely, a total of five drugs corresponding to eight biomarker–drug pairs identified in our study lacked annotations for PharmGKB PGx levels: one of which (umeclidinium/vilanterol-*CYP2D6*) was annotated in a Swissmedic-approved drug label but not in an FDA-approved label, while the remaining seven biomarker–drug pairs (i.e., *TTR*-patisiran, *Deletion 17p*-venetoclax, *FGFR2*-pemigatinib, *ACADVL*-triheptanoin, *CPT2*-triheptanoin, *HADHA*-triheptanoin, *HADHB*-triheptanoin) were not annotated for reasons unknown [59,60,61].

Furthermore, none of the biomarkers identified in our study were assigned to the “recommended genetic testing” category. As of November 10, 2020, PharmGKB listed a total of five drug approvals with “recommended testing”, of which two (i.e., azathioprine and thioguanine) were first approved prior to 2000 and to which biomarker information was added as part of subsequent labeling updates [62]. The remaining three approvals (i.e., dextromethorphan/quinidine, mercaptopurine, and oxcarbazepine) were approved with submission classifications such as Type 4 (new combination), Type 5 (new formulation/new manufacturer), and Type 3 (new dosage form), and were excluded from our analysis. Our findings suggest that there was sufficient evidence at initial approval to warrant testing requirements for relevant drugs, especially for therapies that were developed with indications based on specific genotypes; thus, those were assigned to the “required genetic testing” category instead.

## 5. Conclusions

Advances in genomics research have clearly affected how drugs are developed and approved. Our analysis demonstrates an upward trend in the inclusion of PGx labeling in new drug approvals in the US over the last two decades; the increased trend is more prominent in cancer drugs. Overall, we are likely to see continued growth in new drugs approvals with PGx information. More than half of PGx information in new drug approvals are clinically actionable, with the majority of testing requirements concentrated in cancer drugs. Further studies are warranted to examine the utilization of such tests in clinical practice as well as to generate evidence in support of utilizing PGx biomarkers for non-cancer therapeutic areas.

## Figures and Tables

**Table 1:** Annual proportion of drug approvals with PGx labeling for cancer vs. non-cancer indications.

	All		Cancer		Non-Cancer	
Year	Total Number of New Drugs Approved by FDA	Total No. of New Drugs with Biomarker Mentioned (%)	Number of New Drugs Approved by FDA	New Drugs with Biomarker Mentioned (%)	Number of New Drugs Approved by FDA	New Drugs with Biomarker Mentioned (%)
2000	29	3 (10.3)	3	1 (33.3)	26	2 (7.7)
2001	28	3 (10.7)	2	1 (50.0)	26	2 (7.7)
2002	23	5 (21.7)	4	3 (75.0)	19	2 (10.5)
2003	24	2 (8.3)	3	1 (33.3)	21	1 (4.8)
2004	34	5 (14.7)	5	2 (40.0)	29	3 (10.3)
2005	19	1 (5.3)	3	1 (33.3)	16	0
2006	22	3 (13.6)	5	2 (40.0)	17	1 (5.9)
2007	18	6 (33.3)	4	3 (75.0)	14	3 (21.4)
2008	24	5 (20.8)	3	0	21	5 (23.8)
2009	27	5 (18.5)	5	2 (40.0)	22	3 (13.6)
2010	20	4 (20.0)	2	1 (50.0)	18	3 (16.7)
2011	30	10 (33.3)	7	4 (57.1)	23	6 (26.1)
2012	39	9 (23.1)	12	6 (50.0)	27	3 (11.1)
2013	27	12 (44.4)	9	5 (55.6)	18	7 (38.9)
2014	41	11 (26.8)	8	6 (75.0)	33	5 (15.2)
2015	47	16 (34.0)	14	6 (42.9)	33	10 (30.3)
2016	25	9 (36.0)	4	4 (100.0)	21	5 (23.8)
2017	53	15 (28.3)	14	11 (78.6)	39	4 (10.3)
2018	66	23 (34.8)	18	11 (61.1)	48	12 (25.0)
2019	59	20 (33.9)	18	10 (55.6)	41	10 (24.4)
2020 ^1^	39	11 (28.2)	17	8 (47.1)	22	3 (13.6)

^1^ Data through 31 July 2020.

## Data Availability

The data presented in this study are publicly available and can be downloaded from Drugs@FDA database (https://www.accessdata.fda.gov/scripts/cder/daf/ (accessed on 1 March 2021)) and PharmGKB (https://www.pharmgkb.org (accessed on 1 March 2021)).
